# Supplementary material for: A widespread family of viral sponge proteins reveals specific inhibition of nucleotide signals in anti-phage defense
Source: bioRxiv. 2024 Dec 31:2024.12.30.630793. Preprint. [Version 1] doi: 10.1101/2024.12.30.630793 (PMC11722364; doi:10.1101/2024.12.30.630793)
Supplement: Supplement 2 — Table S2. Crystallographic Statistics, related to Figures 3 and 4 [file media-2.pdf]

Table S2. Crystallographic Statistics, Related to Figures 3 and 4

	***Bacillus* phage SPO1 Acb4–3′3′-cGAMP**
Resolution (Å)[a]	83.73–2.06 (2.17–2.06)
Wavelength (Å)	0.97934
Space group	P 1
Unit cell: a, b, c (Å)	54.28, 56.50, 167.58
Unit cell: α, β, γ (°)	87.53, 89.02, 81.58
Molecules per ASU	20
Total reflections	430,233 (60,909)
Unique reflections	119,318 (17,393)
Completeness (%)[a]	97.8 (97.0)
Multiplicity[a]	3.6 (3.5)
$I/\sigma I$[a]	3.2 (0.6)
CC(1/2)[b] (%)[a]	98.8 (38.9)
Rpim[c] (%)[a]	11.7 (92.2)
Resolution (Å)	83.73–2.06
Free reflections	1,411
R-factor / R-free	21.3 / 25.4
Bond distance (RMS Å)	0.002
Bond angles (RMS °)	0.497
No. atoms: protein	14,462
No. atoms: ligand / ion	900
No. atoms: water	887
Average B-factor: protein	32.62
Average B-factor: ligand	28.79
Average B-factor: water	30.71
Ramachandran plot: favored	99.27%
Ramachandran plot: allowed	0.73%
Ramachandran plot: outliers	0.00%
Rotamer outliers	1.32%
MolProbity[d] score	1.41
Protein Data Bank ID	9E4W

[a] Highest resolution shell values in parentheses
[b] (Karplus and Diederichs, 2012)
[c] (Weiss, 2001)
[d] (Chen et al., 2010)
