# Supplementary material for: A widespread family of viral sponge proteins reveals specific inhibition of nucleotide signals in anti-phage defense
Source: bioRxiv. 2024 Dec 31:2024.12.30.630793. Preprint. [Version 1] doi: 10.1101/2024.12.30.630793 (PMC11722364; doi:10.1101/2024.12.30.630793)

bioRxiv preprint doi: https://doi.org/10.1101/2024.12.30.630793; this version posted December 31, 2024. The copyright holder for this preprint (which was not certified by peer review) is the author/funder, who has granted bioRxiv a license to display the preprint in perpetuity. It is made available under aCC-BY 4.0 International license.

Figure S1. Biochemical screen of 3′3′-cGAMP binding activity in *Bacillus* and *E. coli* phage-infected lysates, related to **Figure 1**

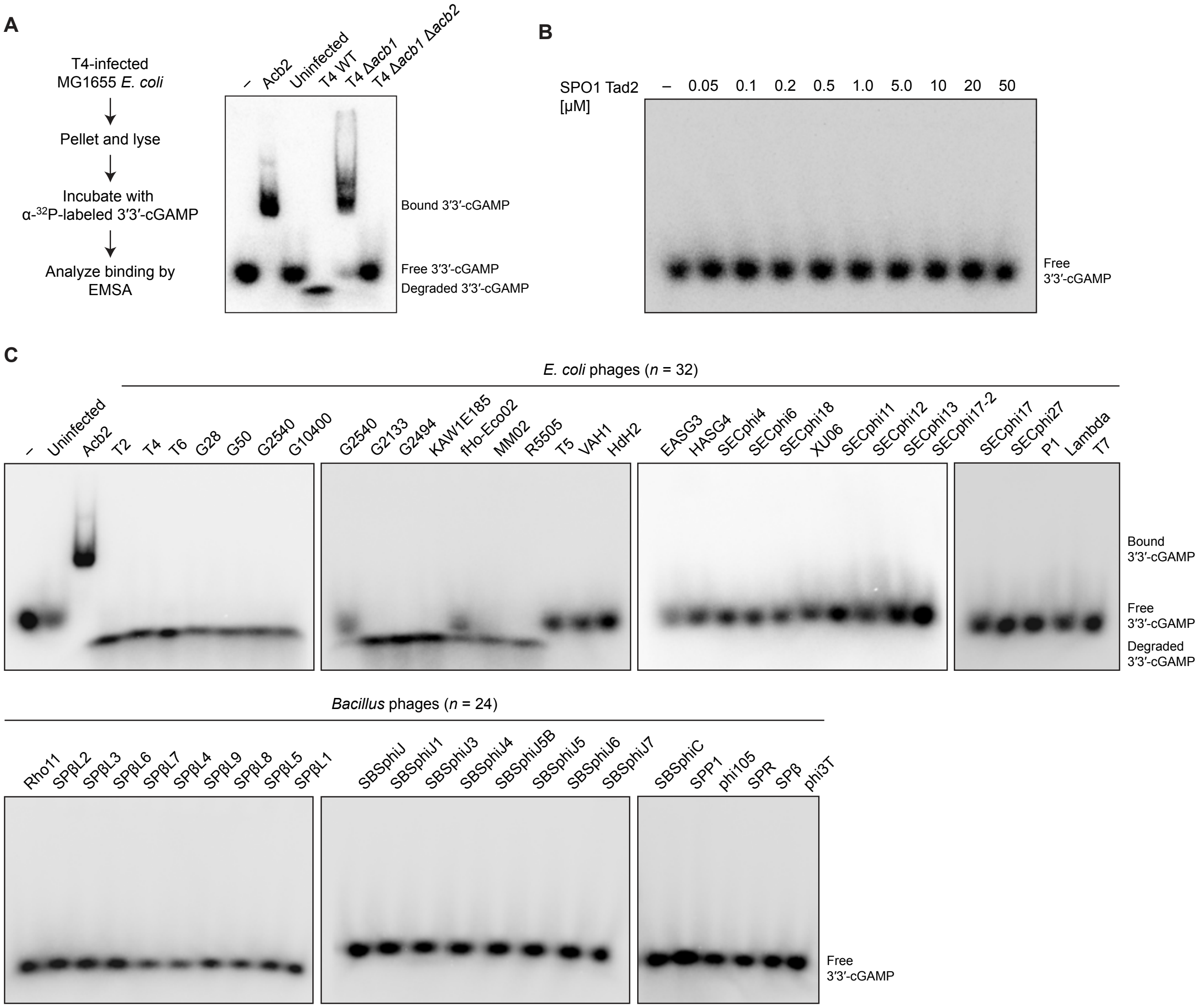

bioRxiv preprint doi: https://doi.org/10.1101/2024.12.30.630793; this version posted December 31, 2024. The copyright holder for this preprint (which was not certified by peer review) is the author/funder, who has granted bioRxiv a license to display the preprint in perpetuity. It is made available under aCC-BY 4.0 International license.

Figure S2. Identification and purification of *Bacillus* phage SPO1 Acb4, related to **Figure 1**

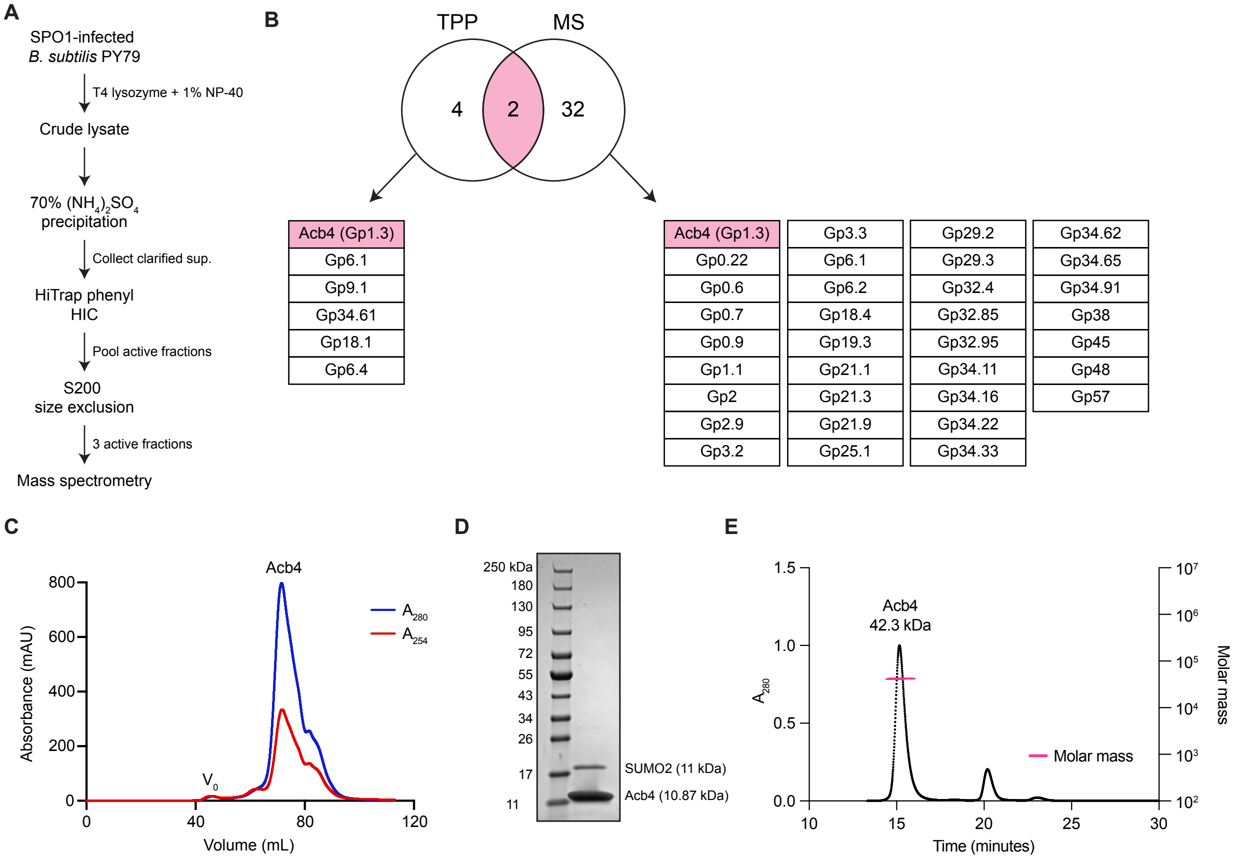

bioRxiv preprint doi: https://doi.org/10.1101/2024.12.30.630793; this version posted December 31, 2024. The copyright holder for this preprint (which was not certified by peer review) is the author/funder, who has granted bioRxiv a license to display the preprint in perpetuity. It is made available under aCC-BY 4.0 International license.

Figure S3. Generation and validation of mutant phage T4 viruses, related to **Figure 2**

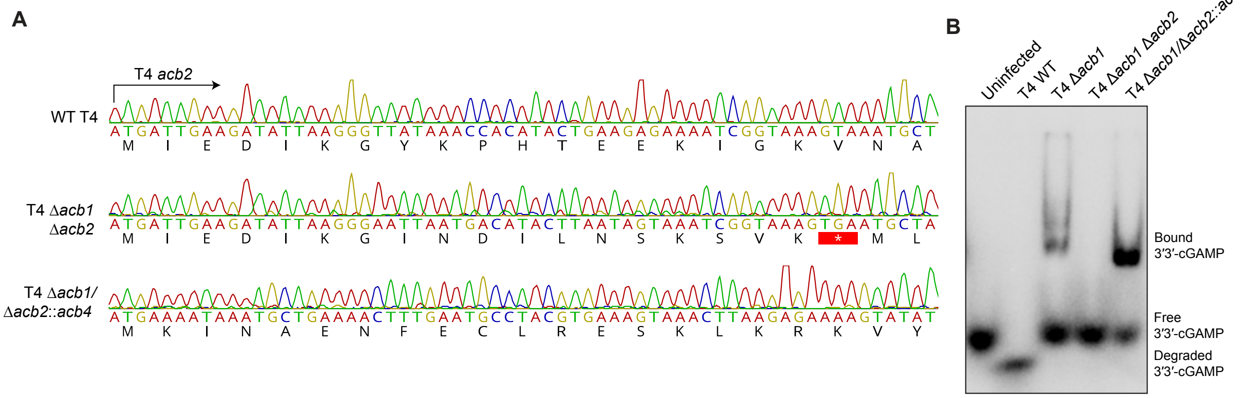

bioRxiv preprint doi: https://doi.org/10.1101/2024.12.30.630793; this version posted December 31, 2024. The copyright holder for this preprint (which was not certified by peer review) is the author/funder, who has granted bioRxiv a license to display the preprint in perpetuity. It is made available under aCC-BY 4.0 International license.

Figure S4. Acb4 structural characterization and diversity of Acb4 homologs, related to **Figures 3–5**

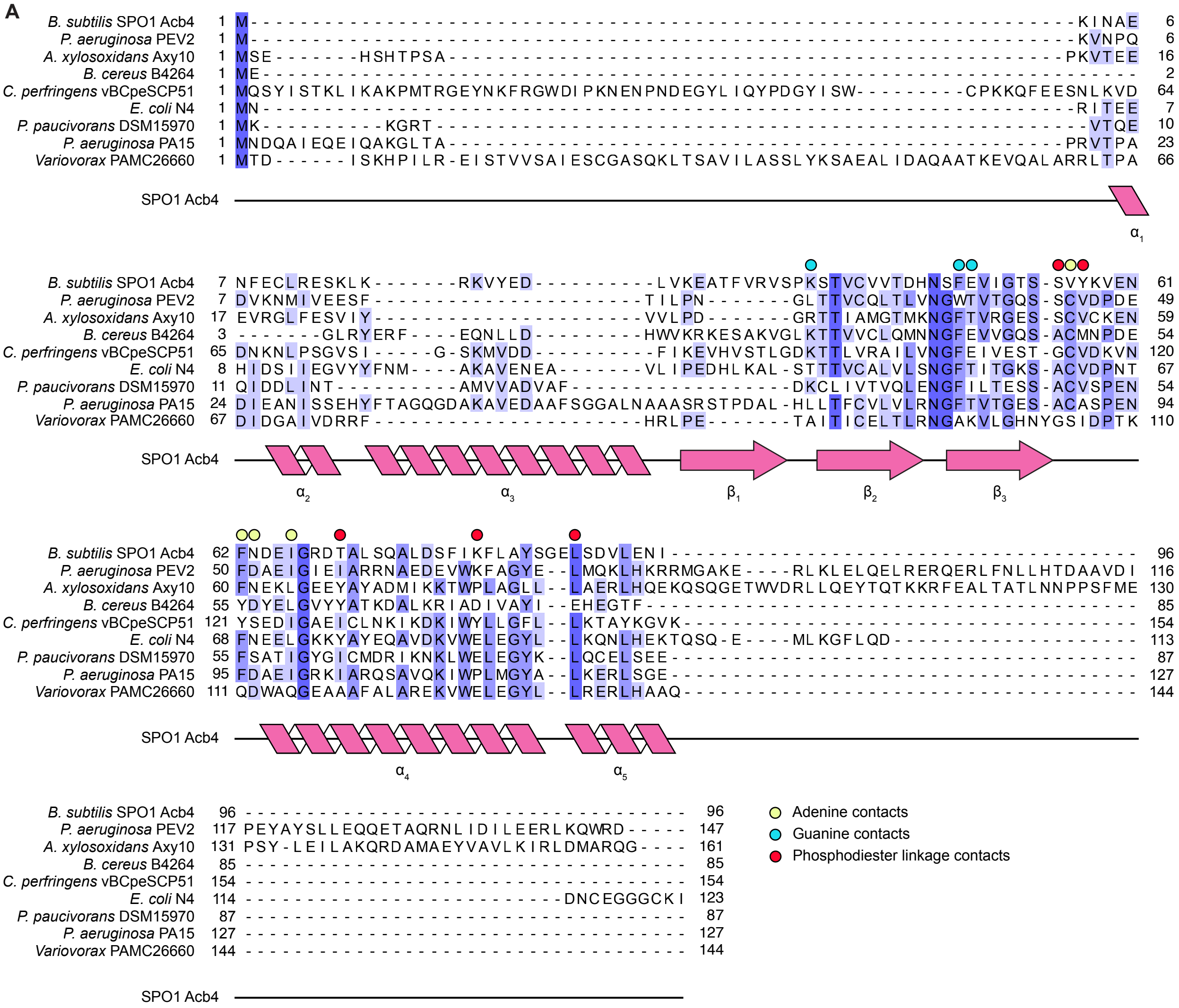

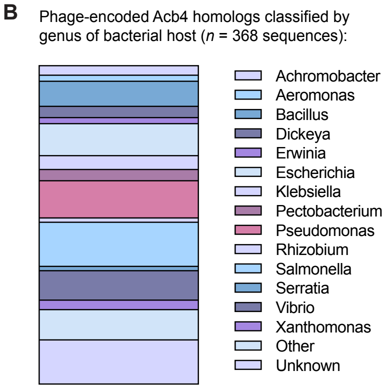

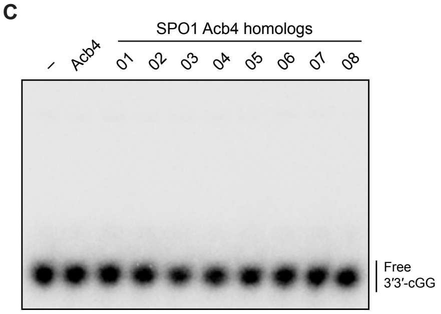

bioRxiv preprint doi: https://doi.org/10.1101/2024.12.30.630793; this version posted December 31, 2024. The copyright holder for this preprint (which was not certified by peer review) is the author/funder, who has granted bioRxiv a license to display the preprint in perpetuity. It is made available under aCC-BY 4.0 International license.

Figure S5. Structural comparison of *Bacillus* phage SPO1 Acb4 with viral sponge families, related to **Figures 3 and 4**

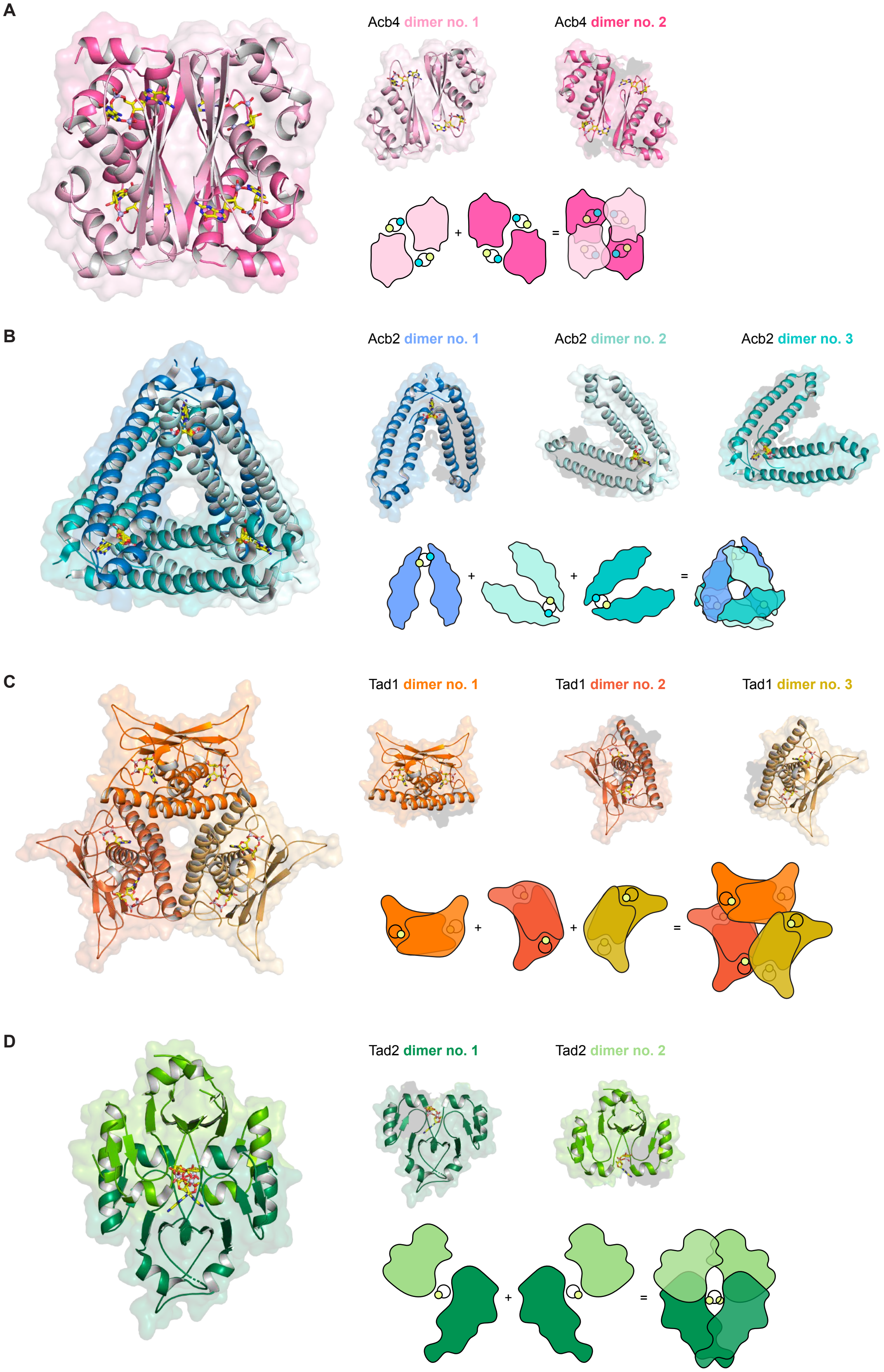

bioRxiv preprint doi: https://doi.org/10.1101/2024.12.30.630793; this version posted December 31, 2024. The copyright holder for this preprint (which was not certified by peer review) is the author/funder, who has granted bioRxiv a license to display the preprint in perpetuity. It is made available under aCC-BY 4.0 International license.

Figure S6. Biochemical and mutagenesis analysis of Acb4 ligand interaction, related to **Figure 4**

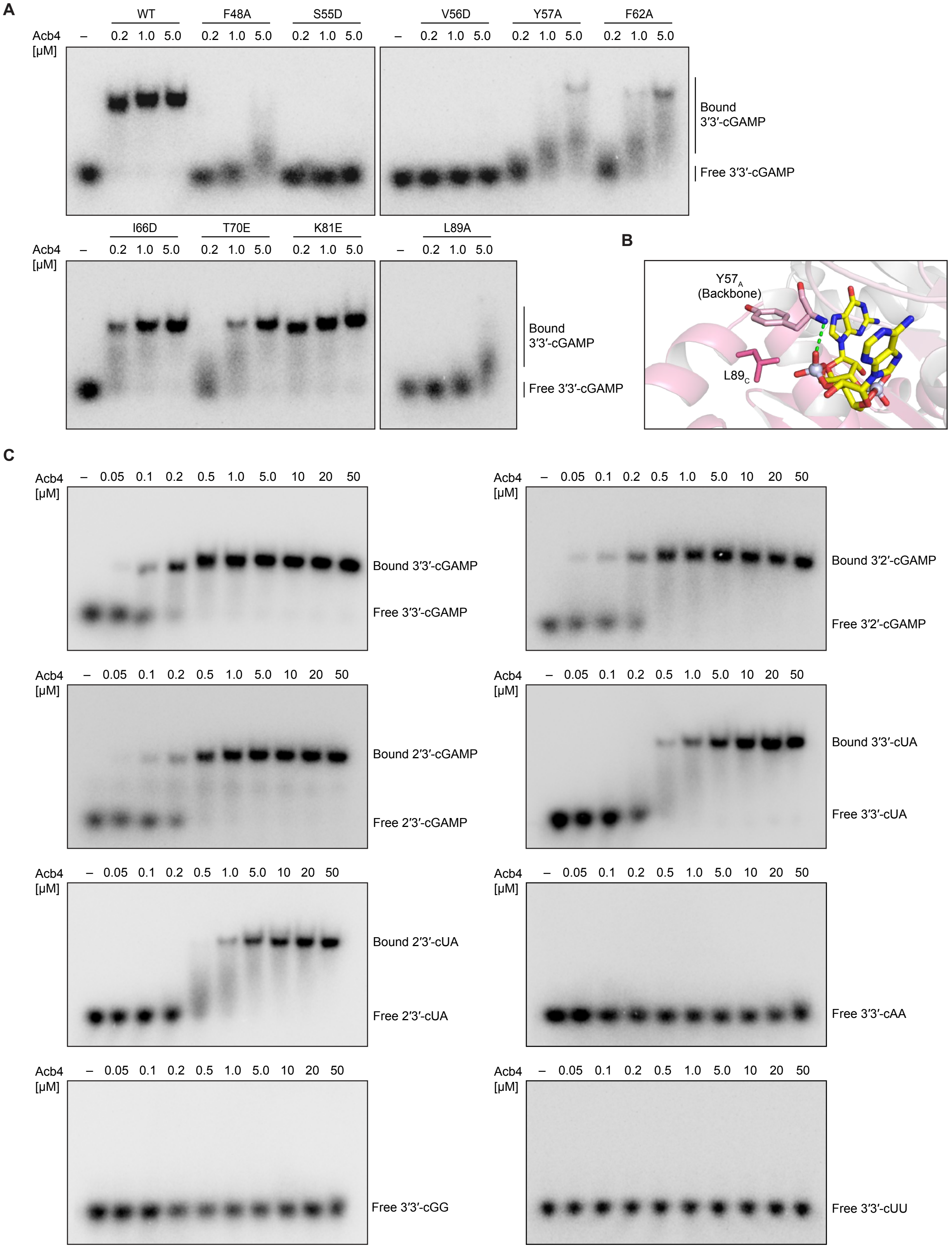

Supplement: 4 — Figure S1. Biochemical screen of 3′3′-cGAMP binding activity in Bacillus subtilis and Escherichia coli phage-infected lysates, related to Figure 1 (A) Schematic of screening approach and EMSA analysis of 3′3′-cGAMP binding and degradation activity detected in wildtype and recombinant T4 phage-infected lysates. Data are representative of at least n = 3 independent experiments. (B) EMSA analysis of the binding of recombinant SPO1 Tad2 and 3′3′-cGAMP. Data are representative of n = 2 independent experiments. (C) Primary data from a biochemical screen for 3′3′-cGAMP binding activity in 32 Escherichia coli phages and 24 Bacillus subtilis phages. Phage-infected lysates were incubated with α32P-radiolabeled 3′3′-cGAMP, and binding or ligand degradation activity was visualized by EMSA. Data are representative of n = 2 independent experiments. For list of phages used in biochemical screen, refer to Table S1. Figure S2. Identification and purification of Bacillus phage SPO1 Acb4, related to Figure 1 (A) Schematic of biochemical fractionation strategy used to enrich 3′3′-cGAMP binding activity from crude Bacillus phage SPO1-infected lysate. (B) Venn diagram comparing candidate Acb4 proteins identified independently through thermal proteome profiling (TPP) and biochemical fractionation coupled with mass spectrometry (MS). (C) Purification of phage SPO1 Acb4 from Escherichia coli. SPO1 Acb4 was expressed as an N-terminal 6×His-SUMO fusion protein and purified by Ni-NTA affinity chromatography followed by S75 size-exclusion chromatography. (D) SDS-PAGE analysis of recombinant Bacillus phage SPO1 Acb4 visualized by Coomassie blue staining. (E) Size-exclusion chromatography with multi-angled light scattering analysis of recombinant Bacillus phage SPO1 Acb4. The Acb4 complex migrates at ~42.3 kDa, consistent with a tetrameric assembly. Figure S3. Generation and validation of mutant phage T4 viruses, related to Figure 2 (A) Sanger sequencing reads confirming successful replacement of [file NIHPP2024.12.30.630793V1-supplement-1.pdf]
